# Supplementary material for: ICIF: Image fusion via information clustering and image features
Source: PLoS One. 2023 Aug 2;18(8):e0286024. doi: 10.1371/journal.pone.0286024 (PMC10396002; doi:10.1371/journal.pone.0286024)
Supplement: S2 File — (DOCX) [file pone.0286024.s002.docx]

**Journal Requirements:**

Please review your reference list to ensure that it is complete and correct. If you have cited papers that have been retracted, please include the rationale for doing so in the manuscript text, or remove these references and replace them with relevant current references. Any changes to the reference list should be mentioned in the rebuttal letter that accompanies your revised manuscript. If you need to cite a retracted article, indicate the article’s retracted status in the References list and also include a citation and full reference for the retraction notice.1

Our actions：Through checking the references, the above problems are not found.

**Reviewer #1:**

**Reviewer #2:** This paper is written very well, but I have few comments for the author

1. Please check your equation format. For example, Equation 14. It has ". I Bmax" and "I Bmin". Please check the difference.

Our actions：We drew inferences from one example and checked all the equations. And correct the problematic equation.

|  | $I_{BF}=\frac{\text{min}(\sigma_{B\text{max}},\sigma_{B\text{min}})}{\sigma_{B\text{max}}+\sigma_{B\text{min}}}\cdot I_{B\text{max}}+(\frac{\text{max}(\sigma_{B\text{max}},\sigma_{B\text{min}})}{\sigma_{B\text{max}}+\sigma_{B\text{min}}})I_{B\text{min}}$, | (14) |
| --- | --- | --- |

instead

|  | $I_{BF}=\frac{\text{min}(\sigma_{B\text{max}},\sigma_{B\text{min}})}{\sigma_{B\text{max}}+\sigma_{B\text{min}}}I_{B\text{max}}+(\frac{\text{max}(\sigma_{B\text{max}},\sigma_{B\text{min}})}{\sigma_{B\text{max}}+\sigma_{B\text{min}}})I_{B\text{min}}$, | (14) |
| --- | --- | --- |

2. Please check your results and discussion. Windows 10 OS, 2.60GHz CPU and 8 GB RAM. What about processor and core ? It is also important.

Our actions：We have supplemented this part of information as follows:

The experiment was conducted on Windows 10 OS, Intel(R) Core(TM) i7-6700HQ CPU @ Dual-Core 2.60GHz, and 8GB RAM, and all the algorithms were implemented with MATLAB2016a.

3. Please check your Table format. For instance, Table 1 size.

Our actions：We checked the table, and found an abnormal length of Table 1, and we reworked Table 1

| SD\Image | Eyeball | |  | Brain | |  | Forest | |  | Camouflage car | |
| --- | --- | --- | --- | --- | --- | --- | --- | --- | --- | --- | --- |
|  | Visible1 | Visible2 |  | CT | MIR |  | Visible | Infrared |  | Visible | Infrared |
| Source map$\sigma$ | 55.83 | 54.64 |  | 30.92 | **22.269** |  | **20.45** | 30.32 |  | 42.83 | 59.81 |
| Grassroots$\sigma$ | 53.83 | 52.83 |  | **11.49** | **6.961** |  | **0.32** | **14.59** |  | **28.78** | 31.92 |

instead

| SD\Image | Eyeball | | Brain | | Forest | | Camouflage car | |
| --- | --- | --- | --- | --- | --- | --- | --- | --- |
|  | Visible1 | Visible2 | CT | MIR | Visible | Infrared | Visible | Infrared |
| Source map$\sigma$ | 55.83 | 54.64 | 30.92 | **22.269** | **20.45** | 30.32 | 42.83 | 59.81 |
| Grassroots$\sigma$ | 53.83 | 52.83 | **11.49** | **6.961** | **0.32** | **14.59** | **28.78** | 31.92 |

4. Please discuss more about which database you have used.

Our actions：We introduce the TON database and present links to the database with the aim of improving the availability of data, which are also commonly used by other scholars.

The TNO dataset contains multispectral (such as enhanced vision, near-infrared and long-wave infrared, and thermal) nocturnal images of different military-related scenes. The TNO dataset contains 60 pairs of infrared and visible light images and three sequences involving 19, 32, and 23 image pairs, respectively. We selected Seven of the most challenging and commonly used images (by other researchers) for qualitative evaluation of objects, (http://figshare.com/articles/TNO_Image_Fusion_Dataset/1008029).

Also, experiments on the other sensor fusions were conducted to verify the generalization ability of the proposed ICIF algorithm(http://www.med.harvard.edu/AANLIB/home.html). The fusion results are shown in Fig 11
